# An Activation Likelihood Estimation Meta-Analysis of Specific Functional Alterations in Dorsal Attention Network in Mild Cognitive Impairment

**DOI:** 10.3389/fnins.2022.876568

**Published:** 2022-04-26

**Authors:** Huimin Wu, Yu Song, Shanshan Chen, Honglin Ge, Zheng Yan, Wenzhang Qi, Qianqian Yuan, Xuhong Liang, Xingjian Lin, Jiu Chen

**Affiliations:** ^1^Department of Neurology, The Affiliated Brain Hospital of Nanjing Medical University, Nanjing, China; ^2^Institute of Neuropsychiatry, The Affiliated Brain Hospital of Nanjing Medical University, Nanjing, China; ^3^Institute of Brain Functional Imaging, Nanjing Medical University, Nanjing, China; ^4^Department of Radiology, The Affiliated Brain Hospital of Nanjing Medical University, Nanjing, China

**Keywords:** mild cognitive impairment, amplitude of low-frequency fluctuation, regional homogeneity, functional connectivity, activation likelihood estimation, dorsal attention network

## Abstract

**Background:**

Mild cognitive impairment (MCI) is known as the prodromal stage of the Alzheimer’s disease (AD) spectrum. The recent studies have advised that functional alterations in the dorsal attention network (DAN) could be used as a sensitive marker to forecast the progression from MCI to AD. Therefore, our aim was to investigate specific functional alterations in the DAN in MCI.

**Methods:**

We systematically searched PubMed, EMBASE, and Web of Science and chose relevant articles based on the three functional indicators, the amplitude of low-frequency fluctuation (ALFF), regional homogeneity (ReHo), and functional connectivity (FC) in the DAN in MCI. Based on the activation likelihood estimation, we accomplished the aggregation of specific coordinates and the analysis of functional alterations.

**Results:**

A total of 38 studies were involved in our meta-analysis. By summing up included articles, we acquired specific brain region alterations in the DAN mainly in the superior temporal gyrus (STG), middle temporal gyrus (MTG), superior frontal gyrus (SFG), middle frontal gyrus (MFG), inferior frontal gyrus (IFG), precentral gyrus (preCG), inferior parietal lobule (IPL), superior parietal lobule (SPL). At the same time, the key area that shows anti-interaction with default mode network included the IPL in the DAN. The one showing interactions with executive control network was mainly in the MFG. Finally, the frontoparietal network showed a close connection with DAN especially in the IPL and IFG.

**Conclusion:**

This study demonstrated abnormal functional markers in the DAN and its interactions with other networks in MCI group, respectively. It provided the foundation for future targeted interventions in preventing the progression of AD.

**Systematic Review Registration:**

[https://www.crd.york.ac.uk/PROSPERO/], identifier [CRD42021287958].

## Introduction

Alzheimer’s disease (AD) is one of the most common causes of dementia, which causes degeneration of the cells in the brain. The decline of reflection and independence in personal daily living ability is evident in the progress of the disease (Vaz and Silvestre, 2020). Unfortunately, there are no effective treatment options for AD with massive research (Yiannopoulou and Papageorgiou, 2020). Mild cognitive impairment (MCI) is known as the prodromal stage of the AD spectrum. People with MCI can show cognitive function not normal for age and decline in cognition, essentially normal functional activities, and without dementia (Kantarci et al., 2009). We further discussed specific functional changes of MCI groups, which can provide targets for the early intervention in the progression of AD.

The resting-state functional magnetic resonance imaging (rs-fMRI) is an essential auxiliary diagnostic method to detect some changes in functional brain networks (Liang et al., 2020). One such advance is the amplitude of low-frequency fluctuation (ALFF), which is thought to reflect the spontaneous activity of neurons. A high value indicates that the neurons in this brain area are active (Cai et al., 2017). Another measure is regional homogeneity (ReHo), which reflects the consistency of neuronal activity in local brain areas (Cha et al., 2015). The increased value indicates that the neuronal activity in the local brain area tends to increase. The third measure is functional connectivity (FC), which represents the neurophysiological activity with a certain distance in space (Qian et al., 2015). Thus, the three indicators such as ALFF, ReHo, and FC can locally reveal the consistency of neuronal activity and comprehensively show the connections of brain regions and networks.

Recently, some studies have shown specific functional alternations in the default mode network (DMN; Yuan et al., 2021), salience network (SN; Song et al., 2021), and executive control network (ECN; Xu et al., 2020) in the patients with MCI. Dorsal attention network (DAN) has been related to working memory and episodic memory which play an essential role in cognitive function. It is mainly responsible for the “top-down” attention process and keeps us focused (Zhan et al., 2016). The recent studies have advised that functional alterations in the DAN could be used as a sensitive marker to forecast the progression from MCI to AD (Qian et al., 2015). However, adequate image data are lacking to find specific functional changes in the DAN. DAN mainly employed in the intraparietal sulcus (IPS) and superior or middle frontal gyrus or precentral gyrus (FEF area), which contributes to the process of goal and selection of stimuli (Fox et al., 2006). In addition, multiple studies have examined the relationships between DAN and other networks. Prior studies have reported a decreased anticorrelation between the DMN and the DAN in MCI (Wang J. et al., 2019). However, there was insufficient data to find reliable specific imaging markers in the DAN to reflect the relationship between DAN and other networks. Thus, summarizing specific functional changes of the DAN and exploring its interactions with other networks can be essential.

One of the most commonly used algorithms for coordinate-based meta-analysis is activation likelihood estimation (ALE; Eickhoff et al., 2012). Instead of treating activation points in neuroimaging studies as single activation points, ALE treats each equilibrium activation peak as having a three-dimensional Gaussian probability density function centered at given coordinates and draws an ALE map (Laird et al., 2005). ALE determines whether there are anatomical or functional differences and convergence between human brain imaging studies based on the multiple coordinates. It has been widely used in rs-fMRI studies (Zhang et al., 2012). The advantage of the ALE technique is that it uses the coordinates of the abnormal anatomical site rather than the labels, thereby avoiding the drawbacks. Another benefit of this technique is excluding negative data from the results (Murphy et al., 2003). Therefore, we use ALE to output the results by inputting aggregated DAN coordinates from independent experiments. A study by Gu and Zhang (2019) obtained key regions related to gray matter atrophy in MCI using ALE and suggested that regional alternations might act as the diagnostic biomarkers. However, this study was the first one to access functional specific changes in the DAN in patients with MCI.

Hence, the study aims to explain (1) comprehensively abnormal markers in the DAN in patients with MCI (2) the interactions of specific brain regions in the DAN with other networks.

## Materials and Methods

### Search Strategy

We systematically and comprehensively searched EMBASE, PubMed, and Web of Science. The search terms were as follows: (1) (“functional magnetic resonance imaging” [MeSH] OR (resting state)) AND (“mild cognitive impairment” [MeSH]) AND [(DAN) OR (attention network)] AND [(functional connectivity) OR (FC)]. (2) (“functional magnetic resonance imaging” [MeSH] OR (resting state)) AND (“mild cognitive impairment” [MeSH]) AND [(regional homogeneity) OR (ReHo)]. (3) (“functional magnetic resonance imaging” [MeSH] OR (resting state)) AND (“mild cognitive impairment” [MeSH]) AND [(amplitude of low frequency fluctuation) OR (ALFF)].

### Inclusion and Exclusion Criteria

Our entry criteria were included (1) reported significant alterations of ALFF, ReHo, or FC in the DAN, (2) made comparisons between MCI and healthy control (HC), (3) reported information about the space in Talairach or Montreal Neurological Institute (MNI) coordinates, and (4) were published in English in peer-reviewed journals.

The patients with MCI met the following criteria: (a) attention to cognitive change, (b) impairment of one or more cognitive domains, (c) maintain functional independence in daily life, and (d) not demented (Albert et al., 2011).

Exclusion criteria were as follows: (1) patients were diagnosed with other disease such as Parkinson’s disease, (2) meta-analysis, review, and case report, (3) a lack of regular control group or comparison related coordinates.

**FIGURE 1:**
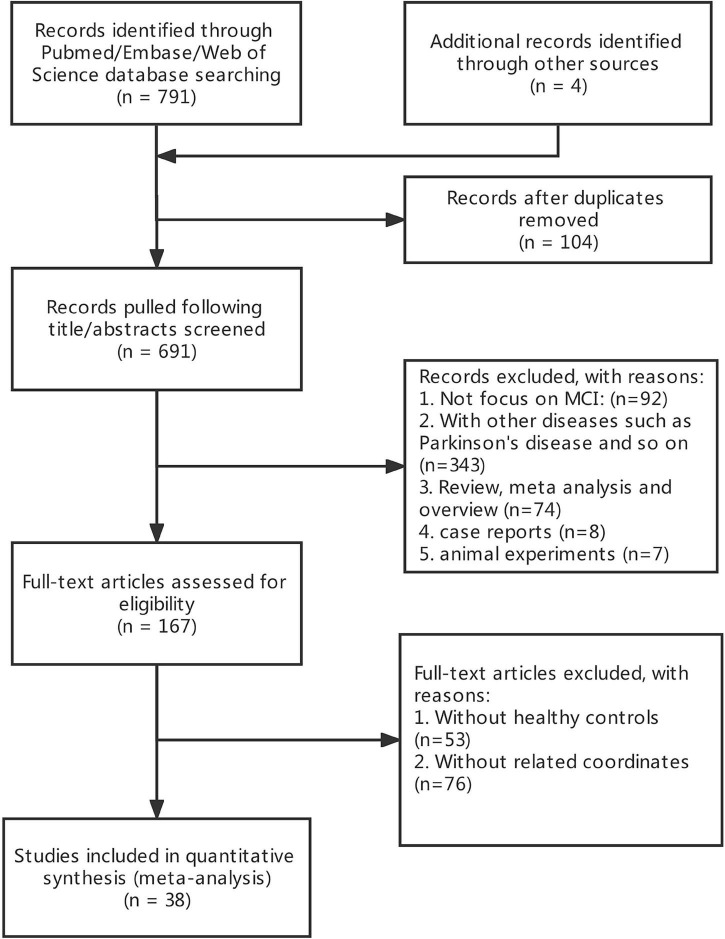
Flow chart showing study selection process.

A total of 691 publications were initially retrieved. After careful screening, a total of 38 publications were included in the final analysis (Figure 1). The included studies met the criteria of 38 MCI (17 ALFF, 14 ReHo, and 7 FC).

### Data Extraction and Quality Assessment

The two researchers in our group extracted data from the literature. First, we included patients with MCI with the specific criteria. Second, we read each study to determine whether it had healthy control group or comparison related coordinates. Then, whether it was a study of ALFF, ReHo, or FC in the DAN. Finally, we extracted coordinates of the DAN in the literature and worked with the method in the form of MNI coordinates. If two current researchers disagree on the adoption of the article, the third researcher will vote on the decision.

### Data Analysis Procedures

We divided MCI subjects into increased and decreased groups on three indexes (ALFF, ReHo, and FC): (1) increased ALFF (321 subjects, 22 foci, and 13 experiments); decreased ALFF (201 subjects, 26 foci, and 9 experiments); (2) increased ReHo (216 subjects, 13 foci, and 7 experiments); decreased ReHo (344 subjects, 29 foci, and 12 experiments); (3) increased FC (115 subjects, 12 foci, and 5 experiments); decreased FC (77 subjects, 7 foci, and 3 experiments).

**FIGURE 2:**
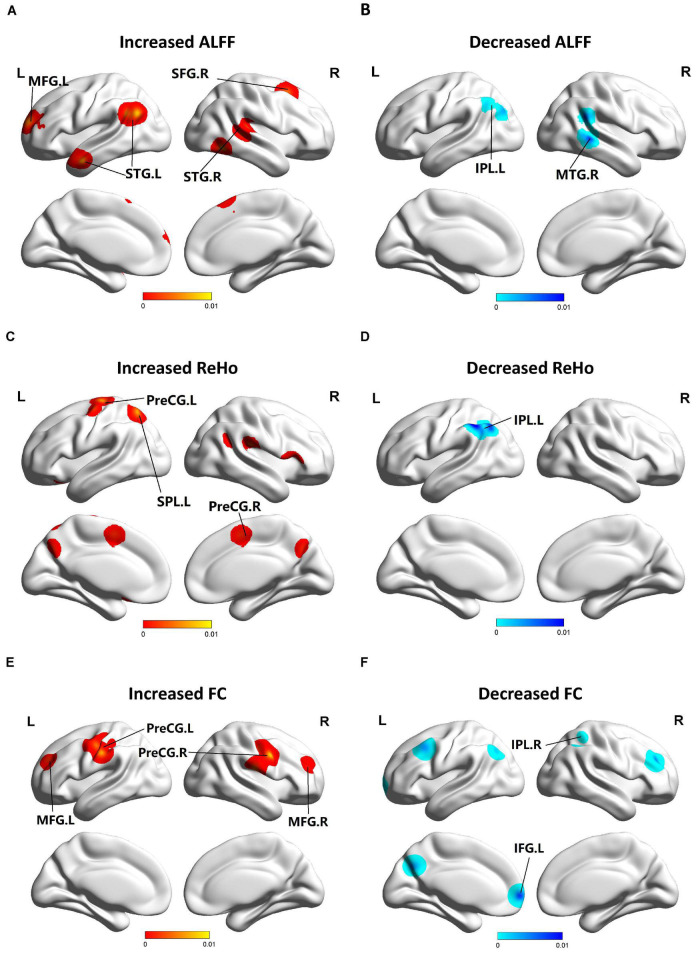
Functional changes of patients with MCI compared with HCs. **(A)** Brain regions showing increased ALFF in patients with MCI compared with HCs. **(B)** Brain regions showing decreased ALFF in patients with MCI compared with HCs. **(C)** Brain regions showing increased ReHo in patients with MCI compared with HCs. **(D)** Brain regions showing decreased ReHo in patients with MCI compared with HCs. **(E)** Brain regions showing increased FC in patients with MCI compared with HCs. **(F)** Brain regions showing decreased FC in patients with MCI compared with HCs. The blue parts indicate the decreased changes, and areas with increased change are displayed in red.

This study used Java-based version of Ginger ALE 2.3.6^1^ to assess the junction of the difference between MCI and HC group in terms of foci across the studies (Eickhoff et al., 2012). We format the collected foci which were performed in MNI coordinates into six text files. We imported them into the software by setting a threshold at *p* < 0.05. The ALE map was performed with a cluster-level family-wise error (FWE) correction at *p* < 0.05 and 1,000 threshold permutations. The FWE correction threshold is set to an ALE value that does not exceed this value for the specified portion of the distribution. FWE thresholds are conservative, so a 5% randomized study or *p* < 0.05 is recommended (Eickhoff et al., 2012). The maps were covered into MNI152 and visualized with the BrainNet Viewer^2^ (Xia et al., 2013) in the Matlab R2013b. The results are shown in Figure 2. The meta-analysis was registered in advance on PROSPERO (registration number: CRD42021287958).^3^

## Results

### Search Results

The study characteristics and results are summarized in Table 1.

**TABLE 1:** The characteristics and results are summarized in the meta-analysis.

References	Sample size (n)	Gender (M/F)	Age (SD)	MMSE (SD)	Group contrasts	Foci (n)	Threshold
**ALFF**							
Zhuang et al. (2020)	MCI 43	0/17	60.71 (6.32)	–	MCI < HC	0	*P* < 0.05
		18/8	64.50 (5.64)				
	HC 29	1/14	58.20 (4.92)		MCI > HC	2	
		6/8	66.79 (3.68)				
Jia et al. (2015)	MCI 7	–	74.1 (7.8)	27.0 (2.3)	MCI < HC	1	*P* < 0.01
	HC 15		70.2 (7.1)	29.2 (1.3)	MCI > HC	1	
Wang et al. (2011)	MCI 16	7/9	69.38 (7.00)	26.50 (1.03)	MCI < HC	5	*P* < 0.05
	HC 22	7/15	66.55 (7.67)	28.59 (0.59)	MCI > HC	2	
Han et al. (2012)	MCI 17	7/10	69.7 (7.6)	25.2 (3.5)	MCI < HC	3	*P* < 0.01
	HC 18	7/11	66.5 (6.2)	29.2 (0.7)	MCI > HC	1	
Cai et al. (2017)	MCI 39	19/20	72.4 (5.01)	25.51 (2.88)	MCI < HC	0	*P* < 0.05
	HC 38	19/19	73.92 (3.90)	29.28 (0.88)	MCI > HC	4	
Cha et al. (2015)	MCI 34	18/16	68.4 (7.9)	27.1 (2.1)	MCI < HC	7	*P* < 0.05
	HC 62	17/45	68.5 (8.0)	28.6 (1.9)	MCI > HC	0	
Zhao et al. (2014)	MCI 20	8/12	65.11 (9.92)	25.21 (2.24)	MCI < HC	4	*P* < 0.05
	HC 18	8/10	66.81 (7.43)	29.31 (1.22)	MCI > HC	2	
Zhuang et al. (2012)	MCI 47	28/19	71.957 (4.777)	26.979 (1.525)	MCI < HC	1	*P* < 0.05
	HC 33	18/15	72.848 (3.392)	28.182 (1.334)	MCI > HC	0	
Zhuang et al. (2019)	MCI 35	16/8	70.42 (4.39)	27.04 (1.49)	MCI < HC	0	*P* < 0.05
		7/4	71.91 (4.39)	27.64 (1.36)			
	HC 26	11/5	70.06 (6.58)	28.31 (1.40)	MCI > HC	1	
		5/5	67.80 (2.39)	27.90 (1.29)			
Zhou et al. (2020)	MCI 47	13/10	70.4 (8.3)	24.7 (3.7)	MCI < HC	0	*P* < 0.05
		10/14	69.8 (6.2)	23.9 (3.6)	MCI > HC	3	
	HC 32	14/18	67.9 (6.4)	28.0 (1.9)			
Xi et al. (2012)	MCI 18	8/10	67.28 (7.87)	24.77 (3.84)	MCI < HC	0	*P* < 0.01
	HC 20	9/11	64.65 (5.59)	28.23 (1.77)	MCI > HC	1	
Hu et al. (2021)	MCI 32	19/13	75.38 (7.91)	28.88 (1.36)	MCI < HC	0	*P* < 0.05
	HC 37	23/14	73.38 (7.00)	29.11 (0.97)	MCI > HC	1	
Wang P. et al. (2019)	MCI 17	9/8	70.53 (4.54)	24.47 (3.88)	MCI < HC	1	*P* < 0.001
	HC 16	8/8	68.56 (5.76)	28.25 (1.39)	MCI > HC	0	
Mascali et al. (2015)	MCI 10	6/4	70.7 (7.1)	25.8 (2.3)	MCI < HC	0	*P* < 0.05
	HC 10	7/3	66.0 (9.6)	29.30 (0.67)	MCI > HC	2	
Yin et al. (2014)	MCI 11	2/9	66.6 (8.7)	24.6 (3.2)	MCI < HC	2	*P* < 0.05
	HC 22	12/10	62.1 (8.1)	29.2 (1.1)	MCI > HC	1	
Ni et al. (2016)	MCI 26	12/14	71 (9)	25 (1.48)	MCI < HC	0	*P* < 0.05
	HC 28	17/11	70 (9)	29 (1.09)	MCI > HC	1	
Liu et al. (2013)	MCI 32	16/16	74.91 (5.88)	–	MCI < HC	2	*P* < 0.05
	HC 28	15/13	77.30 (7.33)		MCI > HC	0	
**ReHo**							
Wang et al. (2015)	MCI 30	18/12	69.1 (5.8)	26.2 (2.2)	MCI < HC	3	*P* < 0.01
	HC 32	15/17	70.1 (5.5)	28.1 (1.5)	MCI > HC	0	
Liu et al. (2014)	MCI 12	1/11	59.3 (3.3)	26.4 (0.9)	MCI < HC	1	*P* < 0.01
	HC 12	4/8	60.6 (5.8)	29.8 (0.4)	MCI > HC	5	
Cai et al. (2018)	MCI 50	24/26	72.3 (6.86)	24.3 (2.45)	MCI < HC	1	*P* < 0.01
	HC 53	29/24	76.08 (6.45)	28.2 (2.13)	MCI > HC	0	
Cha et al. (2015)	MCI 34	18/16	68.4 (7.9)	27.1 (2.1)	MCI < HC	4	*P* < 0.05
	HC 62	17/45	68.5 (8.0)	28.6 (1.9)	MCI > HC	0	
Zhang et al. (2010)	MCI 48	30/18	72.04 (4.42)	27	MCI < HC	4	*P* < 0.05
	HC 36	17/19	71.64 (3.72)	29	MCI > HC	1	
Min et al. (2019)	MCI 10	5/5	69.80 (2.658)	25.90 (0.738)	MCI < HC	3	*P* < 0.05
	HC 10	5/5	69.90 (2.601)	29.30 (0.823)	MCI > HC	3	
Yuan et al. (2016)	MCI 36	17/19	66.8 (9.5)	24.9 (3.4)	MCI < HC	1	*P* < 0.001
	HC 46	19/27	64.3 (7.8)	28.5 (2.0)	MCI > HC	1	
Zhang et al. (2021)	MCI 28	13/15	65.71 (6.895)	27	MCI < HC	2	*P* < 0.05
	HC 37	15/22	63.86 (8.250)	29	MCI > HC	0	
Ni et al. (2016)	MCI 26	12/14	71 (9)	25 (1.48)	MCI < HC	0	*P* < 0.05
	HC 28	17/11	70 (9)	29 (1.09)	MCI > HC	1	
Liu et al. (2021)	MCI 28	14/14	68.39 (4.65)	–	MCI < HC	2	*P* < 0.05
	HC 38	18/20	68.66 (5.09)		MCI > HC	0	
Zhang et al. (2012)	MCI 19	10/9	76 (8)	27 (2)	MCI < HC	6	*P* < 0.01
	HC 21	12/9	70 (7)	29 (1)	MCI > HC	0	
Long et al. (2016)	MCI 29	13/16	66.55 (8.36)	23.38 (3.03)	MCI < HC	1	*P* < 0.01
	HC 33	12/21	62.91 (8.08)	27.94 (1.60)	MCI > HC	0	
Luo et al. (2018)	MCI 64	17/15	72.43 (4.25)	28.34 (1.68)	MCI < HC	0	*P* < 0.05
		17/15	74.90 (5.27)	27.16 (1.71)	MCI > HC	1	
	HC 49	18/31	73.33 (4.60)	29.02 (1.20)			
Bai et al. (2008)	MCI 20	10/10	71.3 (3.8)	27.2 (1.6)	MCI < HC	1	*P* < 0.05
	HC 20	11/9	69.4 (3.8)	28.3 (1.4)	MCI > HC	1	
**FC**							
Lin et al. (2014)	MCI 30	17/13	70.80 (8.26)	28.23 (1.10)	MCI < HC	0	*P* < 0.05
	HC 30	16/14	69.81 (5.79)	29.29 (0.69)	MCI > HC	1	
Bi et al. (2018)	MCI 38	23/15	72.99 (7.79)	27.11 (2.44)	MCI < HC	2	*P* < 0.05
	HC 32	13/19	76.25 (6.51)	29.13 (1.31)	MCI > HC	0	
Cera et al. (2019)	MCI 16	–	65.2	–	MCI < HC	4	*P* < 0.05
	HC 19		73.6		MCI > HC	2	
Zhu et al. (2016)	MCI 19	7/12	65.7 (10.7)	26.7 (1.6)	MCI < HC	0	*P* < 0.05
	HC 28	11/17	63.8 (6.7)	29.0 (0.8)	MCI > HC	3	
Wang J. et al. (2019)	MCI 51	12/10	71.09 (8.41)	24.45 (4.04)	MCI < HC	2	*P* < 0.05
		15/14	71.21 (6.48)	24.07 (3.47)	MCI > HC	0	
	HC 23	10/13	64.61 (9.32)	28.61 (1.50)			
Qian et al. (2015)	MCI 12	7/5	69.3 (6.7)	23.8 (3.4)	MCI < HC	0	*P* < 0.05
	HC 15	8/7	67.8 (7.4)	28.7 (1.2)	MCI > HC	4	
Dannhauser et al. (2005)	MCI 10	5/5	72 (7.7)	24.5 (1.5)	MCI < HC	1	*P* < 0.05
	HC 10	4/6	68 (13.5)	28.3 (1.6)	MCI > HC	0	

The brain areas of the DAN were summarized as follows: (1) use independent component analysis (ICA): inferior occipital gyrus, superior occipital gyrus, superior parietal lobule (SPL), inferior temporal gyrus (Zhang et al., 2020), right superior/middle frontal gyrus (S/MFG), right inferior parietal lobule (IPL), left precentral gyrus (preCG), left IPL/angular gyrus (Qian et al., 2015), dorsolateral prefrontal cortex (dlPFC) and SPL (Avelar-Pereira et al., 2017), IPS, middle temporal gyrus (MTG; Ding et al., 2019), inferior precentral sulcus (Baggio et al., 2015), and ventral IPS (Lei et al., 2014); (2) use seed ROI: MTG, SPL (Liang et al., 2014), dorsal anterior cingulate cortex (dACC; Esposito et al., 2018).

### Meta-Analysis Results

Compared to HCs, patients with MCI showed increased ALFF in the left MFG (BA 9), right SFG (BA 6), left superior temporal gyrus (STG) (BA 39), and right STG (BA 41). Patients with MCI showed decreased ALFF in the left IPL (BA 40) and right MTG (BA 22, 21). Patients with MCI showed increased ReHo in the left preCG (BA 4), left SPL (BA 7), and right preCG (BA 44). Patients with MCI showed decreased ReHo in the left IPL (BA 39, 40). Patients with MCI showed increased FC in the left, right preCG (BA 6) and left, right MFG (BA 9). Patients with MCI showed decreased FC in the left inferior frontal gyrus (IFG) (BA 9) and right IPL (BA 40) (Figure 2).

More details about clusters from the ALE analysis are summarized in Table 2.

**TABLE 2:** Regions with functional changes (ALFF, ReHo, and FC) between MCI and HC.

Cluster	Volume (mm^3^)	MIN			Maximum ALE value	*Z* value	Side	Anatomical regions	BA
		*X*	*Y*	*Z*					

ALFF									
**MCI > HC**									
1	20136	−26	40	16	0.007188175	3.471825	L	Middle frontal gyrus	9
2	16336	−20	12	52	0.007414909	3.6231763	L	Middle frontal gyrus	9
2	16336	4	22	66	0.004700623	2.996591	R	Superior frontal gyrus	6
3	14272	−54	−54	28	0.008169615	3.851295	L	Superior temporal gyrus	39
4	14272	50	−34	10	0.008036611	3.8309553	R	Superior temporal gyrus	41
**MCI < HC**									
1	12840	−42	−58	38	0.009380546	3.9003599	L	Inferior parietal lobule	40
2	11080	60	−42	0	0.008404313	3.7520359	R	Middle temporal gyrus	22
2	11080	62	−48	6	0.00813587	3.4511428	R	Middle temporal gyrus	21

**ReHo**									

**MCI > HC**									
1	11792	−30	−18	69	0.00021037474	1.8419702	L	Precentral gyrus	4
2	7928	−24	−63	57	0.0067398977	3.4334188	L	Superior parietal lobule	7
3	7216	54	18	0	0.0066276384	3.3993435	R	Precentral gyrus	44
**MCI < HC**									
1	19680	−52	−44	44	0.012283053	4.350583	L	Inferior parietal lobule	40
1	19680	−48	−64	42	0.008291758	3.4740293	L	Inferior parietal lobule	39

**FC**									

**MCI > HC**									
1	31088	40	−8	40	0.009056705	4.099734	R	Precentral gyrus	6
1	31088	50	2	34	0.008900939	4.051711	R	Precentral gyrus	6
1	31088	−40	−10	46	0.008636258	3.861686	L	Precentral gyrus	6
2	8792	−33	48	30	0.009058468	4.099734	L	Middle frontal gyrus	9
2	8792	36	51	27	0.008747488	3.9038239	R	Middle frontal gyrus	9
**MCI < HC**									
1	27352	−40	8	26	0.006447163	3.4636831	L	Inferior frontal gyrus	9
2	7568	46	−52	58	0.007144281	3.702397	R	Inferior parietal lobule	40

*BA, Brodmann area; MNI, Montreal Neurological Institute; ALE, activation likelihood estimation; L, left; R, right; MCI, mild cognitive impairment; HC, healthy control.*

## Discussion

This was the first meta-analysis to conduct a comprehensive analysis of all three factors (ALFF, ReHo, and FC) of the DAN in patients with MCI. In our meta-analysis, compared with the healthy group, the specific abnormal brain regions in MCI group were mainly located in the STG, MTG, SFG, MFG, IFG, PreCG, IPL, and SPL.

### Specific Imaging Abnormal Changes in Dorsal Attention Network

The increased ALFF changes showed in the left MFG, right SFG, and STG. A study found that patients with MCI showed increased functional connectivity between the seed regions including bilateral IFG, bilateral MFG, and SFG (Shi et al., 2020). At the same time, the increased changes in the MFG were associated with reduced episodic memory in MCI (Zhao et al., 2019). This can indicate that brain regions that include MFG and SFG with increased ALFF might be closely related to memory loss in the progression of MCI. STG is the key to extract the meaningful language features from the speech input. A recent study also showed increased activity in the STG in MCI, which was consistent with our results (Agosta et al., 2012). To sum up, the increased ALFF in the MFG, SFG, and STG has a certain correlation with language impairment in MCI.

Both the increase and decrease of ALFF indicate the changes in the spontaneous activity of neurons. We described the relationship between the decreased changes of ALFF in specific brain regions and symptoms of patients with MCI below. Right MTG and left IPL demonstrated decreased ALFF changes. The subregions of MTG are related to human episodic memory (Berron et al., 2020). A study indicated that compared with the healthy control group, the stimulation-related activation of MTG in patients with MCI was lower, which is consistent with the indicator’s decline. It is suggested that the attention and cognitive control mechanism of patients with CI may be seriously damaged and became the basis of cognitive defects in this clinical group (Staffen et al., 2012). IPL has a close connection with episodic memory. So, reduced IPL activity indicated impaired memory functional system in patients with MCI which can be the critical early marker for prodromal stages (Zhao et al., 2014). Above all, early reduced ALFF in these brain regions in MCI could be associated with early clinical symptoms, such as impaired memory and attention.

Increased ReHo especially showed in the left, right PreCG, and left SPL. The preCG is located at the primary motor cortex. Its mechanism is to initiate the purposeful movement by integrating the information sent by the sensory motor cortex (Bahmani et al., 2019). An article proves that the impairment in cognitive domains such as working memory and behavioral flexibility can be associated with prefrontal cortex (Parnaudeau et al., 2018). Some studies have shown that blueberry-treated participants exhibited increased blood oxygen level-dependent (BOLD) activation in the left preCG during working memory load condition (Boespflug et al., 2018). SPL has been also related to properties that deal with visuospatial and spatial motion (Banaszkiewicz et al., 2021). An rs-fMRI study also showed increased ReHo in part parietal lobes in MCI, consistent with our findings (Min et al., 2019). Thus, we may hypothesize that the elevation of increased ReHo value in the SPL also plays an essential role in the transition process of AD.

Decreased ReHo especially showed in the left IPL. ReHo mainly explored the consistency of neuronal activity in the local brain area. At the same time, there is both decreasing change in the IPL in MCI. A study demonstrated that M50 sensory gating (SG) deficits in the IPL were related to the poorer performance in the immediate recall of logic memory (LM). Obviously, patients with MCI showed lower auditory short-term memory function with the deficit in the IPL in clinical manifestations (Cheng et al., 2020). It can be concluded that decreased ReHo can be related to the clinical manifestations of patients with MCI.

As to the FC, the increased changes in the MFG were associated with reduced episodic memory in MCI which were the same as increased ALFF (Zhao et al., 2019). The increased FC changes in the preCG were the same as the increased ReHo. A study showed that ReHo focused on the consistency of neuronal activity in local brain areas while FC focused on the connective relationship of two brain regions (Zuo et al., 2018). Thus, the increased FC meant that the connectivity between MFG, preCG, and other regions was higher, making up for deficits in MCI. The decreased FC of the IPL and IFG showed the dysfunction of DAN connectivity, which could be a biomarker to suggest the occurrence of MCI. The activation of IFG was essential for residual language function. At the same time, some task-related functional neuroimaging studies indicated MCI-related low activation in the left IFG (Winhuisen et al., 2005). In a word, with the development of the disease, the brain’s normal function was affected, and partial compensation of functional connectivity was necessary.

According to the specific imaging abnormal changes in DAN, transcranial magnetic stimulation (TMS) and other timely interventions can be carried out. During working memory load conditions, blueberry-treated participants exhibited increased BOLD activation in the left preCG and left IPL (Boespflug et al., 2018). At the same time, the effects of exercise and fitness seem to mainly affect brain structures sensitive to neurodegeneration, especially including frontal and parietal regions (Haeger et al., 2019). To carry out early intervention treatment for patients with MCI, we can carry out a practical course of blueberry taking, exercise, and fitness, which can draw from the above schemes.

### Dorsal Attention Network Interactions With Other Networks

Reviewing the results of ALE analysis of the FC in the DAN, interactions of the DAN with DMN, ECN, and frontoparietal network (FPN) had been observed in MCI group. The main functions of the four networks are different, but they all have overlapping regions and co-activation. The key area that shows anti-interaction with DMN included the IPL in the DAN. At the same time, the one that shows interactions with ECN was mainly in the MFG. Finally, the FPN showed a close connection with DAN, especially in the IPL and IFG.

The DMN is a task-negative network associated with deactivating arduous tasks during attention execution (Weiler et al., 2014). The DAN is also called the task-positive network because its central regions are commonly activated when attention and mind control are required. A study suggested that a reduced anticorrelated activity between DMN and DAN was a part of the normal aging process, and that MCI status was associated with more evident inter-network functional connectivity changes (Esposito et al., 2018). So, the decreased FC of the IPL in the DAN in MCI might be interpreted as a compensation mechanism by impairments of IPL. The interaction among networks may be associated with the structural location.

The ECN has an executive function, which includes problem-solving and working memory, and plays a key role in cognitive regulation and sensory information integration (Xu et al., 2020). The ECN, especially the dlPFC, is structurally connected with the frontal cortices. Therefore, the network is well located and can support a wide range of cognitive processes (Chand et al., 2018). A study highlighted the increased resting-state functional connectivity (rsFC) in the ECN and DAN as neuroimaging indications of disease progression in AD (Joshi et al., 2019). At the same time, the DAN in the MFG showing increased FC also supported this conclusion. Apparently, the increased FC was predictive of impaired episodic memory in MCI and may reflect a dysfunctional change within the episodic memory-related neural network (Zhang et al., 2016).

The FPN is involved in top-down attentional control and allocation of available neural resources to important cognitive processes and motor planning and motor execution (Hsu et al., 2019). The FPN comprises of multiple regions spanning the frontal and parietal cortices, which includes IPL, dlPFC, and preCG (Agosta et al., 2012; Liang et al., 2014). In this study, FPN showed that reduced connectivity in IPL and IFG may lead to the dysfunction of logic, regulating behavior, complex planning, and learning, which patients with MCI can exist (Zhao et al., 2019). This further supported the abnormality of FC as a biomarker for monitoring disease progression.

The interactions related to FC between DAN and other networks, such as DMN, ECN, and FPN, were mainly present in the IPL, IFG, MFG, and PreCG. A study showed for the first time that theta and alpha frequency repetitive transcranial magnetic stimulations (rTMSs) were able to modulate FC in DAN. With theta frequency band in left dlPFC, the memory performs better in a sustained attention task (Kazemi et al., 2020). Thus, changes in the interactions with these networks provided targets for early intervention treatment, which delayed the occurrence of MCI.

### Limitations

Although we have acquired valuable outcomes, some details still need improvement. First of all, the subjects’ age, sex, years of education, and other factors are heterogeneous. However, these factors have no practical impact on the results. What is more, given the limited number of studies included in the analysis, the findings from our meta-analysis should be confirmed in the future research. Last but not least, the selection of seed points of DAN is affected by the subjective idea of the operator, and the selection of different seed points will affect the results to a certain extent. The literature on the coordinates of such seed points can enrich our results in some ways.

## Conclusion

By performing the meta-analysis in patients with MCI to identify the functional changes of DAN, we conclude the evidence of particular functional imaging biomarkers and interactions with other networks such as DMN, ECN, and FPN. These findings offer a further understanding of prospective brain alterations and some interventions for prodromal AD in the MCI group. These meaningful interaction networks supply new insight for selecting brain regions to delay the procession of dementia in the future.

## Data Availability Statement

The original contributions presented in the study are included in the article/Supplementary Material, further inquiries can be directed to the corresponding authors.

## Author Contributions

HW, YS, SC, XJL, and JC designed the study. HG, ZY, WQ, QY, and XHL organized and downloaded the data. HW, YS, and SC analyzed the data and drafted the manuscript. XJL and JC modified the article and approved the submission. All authors contributed to the article and approved the submitted version.

## Conflict of Interest

The authors declare that the research was conducted in the absence of any commercial or financial relationships that could be construed as a potential conflict of interest.

## Publisher’s Note

All claims expressed in this article are solely those of the authors and do not necessarily represent those of their affiliated organizations, or those of the publisher, the editors and the reviewers. Any product that may be evaluated in this article, or claim that may be made by its manufacturer, is not guaranteed or endorsed by the publisher.

## Footnotes

1
http://www.brainmap.org/ale

2
http://www.nitrc.org/projects/bnv/

3
https://mstracker.com

## Abbreviations

MCI: mild cognitive impairment
HCs: healthy controls
ALFF: the amplitude of low frequency fluctuation
ReHo: regional homogeneity
MFG: middle frontal gyrus
STG: superior temporal gyrus
SFG: superior frontal gyrus
IPL: inferior parietal lobule
MTG: middle temporal gyrus
PreCG: precentral gyrus
SPL: superior parietal lobule
IFG: inferior frontal gyrus
R: right
L: left.
