# Supplementary material for: An Activation Likelihood Estimation Meta-Analysis of Specific Functional Alterations in Dorsal Attention Network in Mild Cognitive Impairment
Source: Front Neurosci. 2022 Apr 26;16:876568. doi: 10.3389/fnins.2022.876568 (PMC9086967; doi:10.3389/fnins.2022.876568)
Supplement: Supplementary file 1 [file Table_1.DOC]

**The analysis of FC**

1.Connectivity Features for Identifying Cognitive Impairment in Presymptomatic Carotid Stenosis.

**Regions of the DAN**: left frontal eye field (FEF -26, 6, 48)

2.Non-linear ICA Analysis of Resting-State fMRI in Mild Cognitive Impairment.

**Regions of the DAN**: left medial of superior frontal gyrus (SFGmed), right inferior temporal gyrus (ITG).

3.Altered Cingulate Cortex Functional Connectivity in Normal Aging and Mild Cognitive Impairment.

**Regions of the DAN**: inferior parietal sulcus (IPS), frontal eye field (FEF), anterior cingulate cortex (ACC) and bilateral middle temporal gyrus (MidTempG).

4. Changes of intranetwork and internetwork functional connectivity in Alzheimer’s disease and mild cognitive impairment.

**Regions of the DAN**: R-IPS (24, −56, 55) L-IPS (−27, −52, 27)

5. Dysfunctional interactions between the default mode network and the dorsal attention network in subtypes of amnestic mild cognitive impairment

**Regions of the DAN**: the intraparietal sulcus (IPS; MNI coordinates: -34, -38, 44)

6. Functional-structural degeneration in dorsal and ventral attention systems for Alzheimer’s disease, amnestic mild cognitive impairment.

**Regions of the DAN**: right FEF [24, −13, 51] and right IPS [27, −58, 49], left FEF [−25, −12, 55] and left IPS [−22, −68, 46],

7. The functional anatomy of divided attention in amnestic mild cognitive impairment.

**Regions of the DAN:** prefrontal cortex
